# How do health, social care and legal professionals support visa-dependent migrants affected by a terminal illness in the UK: a qualitative interview study

**DOI:** 10.1136/bmjopen-2025-110213

**Published:** 2026-09-24

**Authors:** Tim Sedgley, Joanne Alexander, Aisha Macgregor, Liz Forbat

**Affiliations:** 1 Birmingham City University, Birmingham, UK; 2 Faculty of Health Sciences and Sport, University of Stirling, Stirling, Scotland, UK; 3 Marie Curie, Edinburgh, UK

**Keywords:** Cultural issues, ethical, quality of life, supportive care, palliative care

## Abstract

**Abstract:**

**Objectives:**

To understand barriers faced by migrants with terminal illness in accessing healthcare services and to document the strategies adopted to mitigate problems they experienced.

**Design:**

Inductive thematic qualitative interview study.

**Setting:**

Community and hospice settings in the UK.

**Participants:**

22 people, comprising 14 working within health/palliative care settings, 4 in legal/policy settings and 4 in migrant support, all with experience of supporting people with a terminal illness.

**Results:**

There are substantial and dire consequences for migrants with terminal illness. While patients and professionals struggle to understand migrants’ entitlements, the expense of treatment and/or the prospect of being charged for healthcare deter access. Healthcare charges and discretionary support were variable. Patients risk accruing heavy healthcare costs/debts, and some avoid accessing care altogether for fear of immigration services being alerted, with consequent fear of removal/deportation.

**Conclusions:**

Service commissioners and palliative care practitioners must be cognisant of the complex constellation of circumstances in which migrants live and die. Strategies are needed to enhance knowledge around entitlements in order to widen access and promote equitable access to healthcare for terminally ill migrants.

**WHAT IS ALREADY KNOWN ON THIS TOPIC**

**WHAT THIS STUDY ADDS**

- Healthcare clinicians and lawyers are not confident in knowing people’s rights.
- Terminally ill migrants are fearful of health debts, and this acts as a barrier to seeking appropriate care and treatment.
- Fear of removal/deportation prevents people from accessing healthcare.

**HOW THIS STUDY MIGHT AFFECT RESEARCH, PRACTICE OR POLICY**

- A *practitioner guide* would be useful to signpost organisations, institutions and charities working in the health/palliative care and immigration sectors.
- Sector-specific training is needed to ensure staff understand migrants’ rights and entitlements.
- Organisations supporting migrants should lead research into the experiences of terminal illness, as they are better placed to build the necessary trust and rapport to recruit them.

**STRENGTHS AND LIMITATIONS OF THIS STUDY**

- Depth interviews with a heterogeneous group of practitioners supporting terminally ill migrants.
- Design and conduct informed by three people with lived experience of terminal illness and migration.
- Despite extensive efforts, no people with direct lived experience of migration and terminal illness were recruited.

## Introduction

Access to healthcare is important for people diagnosed with a terminal illness, whichever country they reside in. Yet migrants’ access to appropriate care for such incurable disease from which is expected to lead to death will depend on their visa type and immigration status. Suboptimal and inequitable access is due to well-recognised factors including institutional racism,^1^ primary care refusals to register patients^2^ alongside language barriers and perceived differences in cultural values and mistrust.^3^

A recent systematic review of first generation migrants and terminal illness drew data from 11 countries, illustrating the international concerns and experiences of this marginalised group.^4^ Access to suitable healthcare and the underlying financial precarity of migrants were recurring themes.

In the UK, the context for terminally ill migrants has changed in recent years. The introduction of the Immigration Health Surcharge (IHS) requires migrants (e.g. on work or study visas) to pay a substantial annual fee to use many parts of the National Health Service (NHS)^5^ (ranging from £776 to £1035 per applicant depending on visa type). Certain migrants are exempt, such as people claiming asylum and those entering the UK on work visas in the health/social care field. The introduction of the IHS was a formalisation of the UK’s increasing tightening of immigration, positioning overseas visitors as a threat to the NHS.^6–8^

IHS cost and the financial impact of being unable to work while ill are considerable. Poverty and deprivation have been invisible yet powerful influences over people’s experiences at the end of life.^9–13^ This risk is particularly acute for people from black and minoritised ethnic groups.^14^ Two-fifths of working age people from minority ethnic backgrounds die below the poverty line.^9–11 15^

In the UK, a small number of health services are provided without charge, for example, seeing a general practitioner (GP), accessing emergency services and some palliative care services.^16^ There are no documents codifying precisely what can be provided free for terminally ill migrants by hospices since these are run by the independent/charity sector. Some secondary care is ‘residence-based’ and for those deemed not ordinarily resident (ie, people without long-term settled status) such services are charged to migrants at 150% of the cost.^17^ With the introduction of charges, processes to monitor, grant or deny access to services are also introduced. This locates the NHS as a site where UK borders are enforced, referred to as a site of bordering practices.^17–19^ Yet, many staff responsible for determining eligibility have limited understanding of the rules.^20^ Consequently, errors can occur where eligible people are denied treatment, ineligible people are granted access or people are billed for services which should not incur a charge. Authors of a recent systematic review call for robust evidence to understand the impact of charging on migrants.^20^

The number of people affected by a terminal illness who are in the UK and subject to visa restrictions is not easy to calculate; figures are not produced by palliative care organisations or government agencies.

Long-term trends indicate increasing numbers of people on employment visas within the UK, from across a range of countries including the EU since Brexit. Many work visas are allocated to individuals who belong to a minoritised ethnic group. In the year ending March 2022, 277 069 work visas were granted (including for dependants).^21^ Data on benefits suggest there are around 25 000 working age people in the UK with a terminal illness who are dying in poverty.^22^ The size of overlap across these categories is not known due to lack of reporting.

Practitioners supporting migrants with a terminal illness hold important knowledge about the barriers for this group accessing the support and care needed. This paper draws on qualitative data from a study examining the intersections of migration and terminal illness to explore experiences of accessing healthcare.

## Methodology

This was a qualitative semistructured interview study, guided by the research question: How do health/social care/legal professionals support visa-dependent migrants affected by a terminal illness in the UK?

The project was guided by a patient and public involvement and engagement (PPIE) group and an interdisciplinary steering group. The PPIE group helped design the research study (one was a coinvestigator), reviewed ethics application paperwork, recruitment processes, and data interpretation. They contributed monthly throughout at team meetings.

### Inclusion criteria

1. Health or social care professionals supporting migrants and their family members affected by terminal illness in the UK.
2. Policy/law professionals, such as employees or former employees of the home office or local/national governments, and legal practitioners with experience of terminally ill migrants in the UK.
3. Adults with lived experience of being a migrant and terminal illness, or being a family member of such. Migrants included anyone in the UK without settled/permanent status who were subject to immigration visas. Terminal illness was defined as an incurable disease from which the person was likely to die within a foreseeable period.
4. Able and willing to provide written consent.

Criterion sampling was employed to recruit professionals and individuals with lived experience who met these inclusion criteria.

Language was not a barrier to participation. Information leaflets were developed in partnership with our PPIE group and provided in several languages, and budget was allocated for interpreter support should participants prefer or require communication in a language not spoken by the research team.

### Recruitment

Recruitment was conducted UK-wide, via a wide range of organisations including (but not limited to) third sector supports for migrants/carers/patients, organisations providing financial advice and guidance, solicitors and immigration lawyers, hospices and palliative care providers, faith-based organisations and local authorities. In total, the team contacted approximately 300 organisations, alongside snowballing techniques and social media (Twitter/X/Meta). The PPIE and steering group shared recruitment materials via their networks. Despite extensive efforts, it was not possible to recruit anyone with lived experience to take part in the study.

### Data collection

Interview topic guides (see online supplemental materials) were developed for the participant groups, generated from the extant literature and shared with participants prior to interview. Questions focused on, for example, how professionals and terminally ill migrants/families experienced key concerns, functional and maladaptive service responses, navigating treatment, work and visa requirements, how patients/families manage financially in the face of terminal illness and enablers and barriers to navigating healthcare and immigration law.

Data were collected by (JA and LF), two female postdoctoral qualitative researchers with more than 10 and 25 years of experience, respectively. There was no prior relationship between researchers and participants prior to data collection.

All data collection occurred online, at the request of participants in 2024. All interviews were one-off contacts, save for one follow-up interview, which was conducted at the request of the interviewee who wanted to reflect further on the case they had described as they learnt more about the circumstances and difficulties of terminally ill migrant they were supporting. Interviews were recorded, with written consent, transcribed and anonymised. The interviews ranged from 38 to 84 min, with an average of 61 min per interview; the follow-up interview lasted 25 min. Field notes supplemented recorded interviews but were not formally integrated into analysis. Transcripts were not returned to respondents.

Sample size was based on principles of theoretical sufficiency, aiming for 24 participants in total.^23^

### Analysis

Thematic analysis^24 25^ was used to develop key themes, following the established method of data familiarisation, initial coding (forming the outline coding tree), searching and reviewing themes, then consolidating/refining and naming themes. Three members of the research team (JA, TS and LF) were involved in independently coding transcripts line-by-line and reviewing the other’s codes in NVivo. The team met regularly to discuss analysis and enhance interpretation. Reflexivity was integrated into the approach including researchers’ own experiences of migration, palliative care and work visas. Respondent checking of transcripts was not used, but findings were discussed at various stages of analysis with the steering and PPIE groups.

### Research team reflexivity

The team comprised individuals who currently or have previously lived as migrants (LF, TS), and where all team members identify as politically left leaning. The team are ideologically focused on equalities for marginalised people and motivated to address the financial impact of terminal illness on migrants in the UK.

### Ethics

All participants were offered a £25 gift card and provided with a list of support organisations they could contact or refer their clients to. All interviewees were advised that infractions of visa regulations would not be reported to immigration services.

### Findings

Interviews were conducted with 22 people working with migrants diagnosed with terminal illness (table 1). Six further people contacted the research team expressing an interest in participating, but did not proceed to interview, either declining due to not meeting inclusion criteria or not responding to further contact.

**Table 1:** Participant characteristics

Inclusion category	Role	Gender
Policy/law	Solicitor	Male
	Immigration lawyer	Female
	Resettlement policy officer	Male
	Local authority service lead	Female
Professional supports	Immigration advisor	Female
	Family support social worker (hospice social worker)	Female
	Clinical nurse (hospice nurse)	Female
	Palliative care social worker	Male
	Refugee support case worker	Male
	Family support social worker (hospice social worker)—follow-up Interview	Female
	Social work student (hospice-based)	Female
	Refugee support worker	Female
	Welfare rights case worker	Female
	Refugee support worker	Female
	Palliative care team (n=9) Palliative care consultant × 1 Healthcare assistant × 2 Nurses × 2 Clinical nurse specialists × 4	1× female 1× male, 1× female 1× male, 1× female 4× female

Barriers to healthcare was a recurring thread across the dataset, and constituted four themes in our coding tree which we report here:

- Legal ambiguity and bureaucracy.
- Disparities in implementation and discretionary compassion.
- Bills and debts.
- Fear of surveillance and immigration enforcement.

### Legal ambiguity and bureaucracy

The legal status of terminally ill migrants’ access to healthcare was marked by confusion, uncertainty and complexity. Access was rendered difficult due to complexities of health service organisation and some services being available to all, some dependent on visa status (and payment of the IHS) and others with potential to incur substantial costs.

Frontline NHS workers, social workers, solicitors and hospice teams lacked clarity on eligibility rules, leading to delays, miscommunications and unequal access. This theme demonstrates how the conflation of immigration control with healthcare provision fosters a legal grey area, where rights to care are inconsistently applied or understood:

Often you have to spend a long time trying to say to consultants and GPs etcetera, “no, no, the person is entitled to treatment and this is the visa that we’re working on.” (Immigration Lawyer)

Determining eligibility for free healthcare was complex, even for experienced professionals. The intricacy and opacity of the costing profile in the UK made it hard to predict likely prices:

The complications of the payments and who is eligible for what […] there’s not a calculator for your treatment […] in the States where you pretty much know, you know, deductibles and insurance and it’s going to cost […] There’s not that sort of knowledge at all here in the UK. People are very scared [that they will] perish in the waiting room. (Resettlement Policy Officer)

Clarifying entitlements was not straightforward. For example, migrants on a UK employment/study visa may be unable to attend work/university due to symptom burden and hence become non-compliant with their visa regulations in terms of minimum requirements for work/study. Such cases would require complex legal representation:

We probably need a barrister to see whether there is any entitlement at the point they are not meeting their visa terms, because there is to some degree a, kind of, contract with the Home Office that you have paid the IHS charge, so, I don’t know if you could argue that, that contract should be fulfilled, or if they would say, well, you have breached it by not meeting your visa terms. So, I don’t know how that would work in law. I would definitely be really cautious with clients who weren’t meeting their visa requirements, as to whether they are fully covered. (Solicitor)

Legal representation by a barrister comes at considerable cost, and hence is not necessarily an option open to people at risk of financial precarity. Many terminally ill migrants relied on pro bono work from lawyers. Yet, the choice otherwise is not to access NHS treatment, to be charged at 150% of the cost to receive NHS services, to access private medical care in the UK or to return to their country of origin to access healthcare there. Under financial constraint none of those options may be possible.

It is notable that these quotations all come from professionals immersed in law, policy and welfare. Ambiguity therefore stems not from lack of expertise but excessive complexity. This underlines the difficulty with which practitioners with expertise in healthcare, or migrants themselves, have in navigating rules and regulations.

### Disparities in implementation and discretionary compassion

Access to care is highly variable and discretionary, based both on legal entitlement and individual staff members’ discretion and attitudes. Some clinicians act compassionately, prioritising patient welfare. Others may rigidly enforce policy, especially if they lacked nuanced knowledge which would enable them to find flexibility. This inconsistency leads to inequitable care provision, where luck determines the availability of treatment.

Some patients were explicitly denied access to the NHS, having built up debts which they were unable to pay off. On their behalf, some professionals requested, and had granted, charging exemptions and NHS debt clearance. Yet gaining access to care without payment was patchy and unpredictable. Some patients had treatments reduced or refused:

[One man] was withdrawn from transplant waiting lists because of his immigration status. […] he still needs a transplant but he has lots of other health conditions which are linked to that. And he has medical bills that are sky-high. […] If he goes to one city, they will treat him there, but if he goes to the other city, they won’t treat him there, just because of his immigration status and I guess, because he’s probably 20 [thousand pounds] in debt to the NHS. (Refugee Support Case Worker)

The following interviewee recounts a situation in which the hospital Overseas Team (responsible for inquiries and charges for people not usually resident in the UK) effectively provided a verbal ‘key’ which, if relayed by the patient, would mean treatment incurred no cost to them:

I spoke to our Overseas Team about that [patient] and they were … I just got the feeling that they were going to try and help, rather than go “these are the rules”. It was very much, “okay this is the situation he’s in, if he says this to me it will put him in a better position” […] and set it up for this man and what he could do to ensure that he would get assessed. And I just got a feeling that they actually, for that particular person anyway, wanted to make the system work for the patient, particularly if they were in palliative circumstances. (Palliative Care Consultant)

Other interviewees talked about finding mechanisms to approve fast-track access to necessary services. Such actions privileged humanitarian ethics above bureaucracy:

I think a lot of the time doctors in hospitals just cracked on, they just took the view that they weren’t accounts departments, and their primary reason was to care. (Solicitor)

### Bills and debt

Cost is a major deterrent for terminally ill migrants accessing necessary treatment. The prospect of incurring large debts or unaffordable bills often led to treatment refusal or avoidance. Even where NHS services had payment plans, the emotional and financial toll created a system where palliative care became inaccessible. Neoliberal healthcare logic forced terminally ill migrants to perform cost vs benefit assessments, the genesis of which stems from the perception, rather than the enforceability, of NHS debt. Therefore, regardless of the feasibility of repayment of incurred debt, financial precarity associated with the cost of NHS care, perceived or otherwise, constituted a barrier which culminated in terminally ill migrants avoiding or refusing treatment:

We had a case where a gentleman was diagnosed with […] gastric cancer and was told he wasn’t eligible for chemotherapy treatment, because he was told how much it would cost him. I can’t remember how many thousands of pounds he was told his bill would be. But he said, “well I can’t afford it so I won’t be having it.” (Clinical Nurse Specialist)

Migrants’ legal status directly correlates with healthcare affordability, resulting in exclusionary outcomes. Even when the debt was seen as notional, as the person would die before being able to pay it back, the cost was seen as too great:

Some are not going to be entitled to free NHS care […] they might get treatment and then they’re going to get a huge bill for it of £40,000 or something, which, in a way, is often completely superfluous because there’s absolutely no way they’re going to pay it and if their life expectancy is, you know, around a year or so, it’s very unlikely that the NHS is ever going to get that money back. But I imagine, you know, it adds to the stress of it all for them. (Welfare Right Case Worker)

The threat of high healthcare bills was viewed as very stressful, even if there was no real expectation of paying off any debts incurred due to their short prognosis, and for some this delayed access to treatment. Two nurses spoke to this issue:

then there’s the issue of what happens now with the financial side (Palliative care nurse)

sometimes it’s late presentation when they come to us because they may avoid early intervention. […] we often see people late on or not engaging as much as maybe they could. (Palliative care nurse)

Migrants’ internalised assumptions of fiscal penalty activate an *anticipatory self-exclusionary response*. Subsequently, in parallel with other immigration regulatory practices, the act of self-exclusion exposes migrant billing policies that promulgate financial and psycho-emotional strain and limit access to healthcare.

For some families, repaying the debt was possible when payment terms were negotiated, which reflects the high incomes and proven financial stability migrants often need to earn to warrant being offered a visa. However, other migrant families were willing and able to repay bills that accrued during treatment:

They continued to access the treatment [changing visa type] was a success story and it worked. But they still have to pay and they were happy to pay…the family was quite happy to clear the debts that had been incurred. (Student Social Worker – Hospice Placement)

This runs counter to populist views that migrants are a drain on statutory services, underscores a *dependency–delinquency* rhetoric, and highlights immigration and health policies that are inherently contradictory. Immigration policy preselects migrants using criteria based on economic independence; however, NHS billing policy is built on the assumption that migrants will draw on NHS services without contribution (*dependency*) and, when billed, will not pay (*delinquency*).^26^ Policy measures, such as requirements for advance payment, as seen with the IHS, set the expectation that frontline healthcare workers will act as border control agents and enforced debt collection, are all measures that underpin these assumptions. Furthermore, such policy contradictions perpetuate inequity in terminally ill migrants’ palliative and end-of-life care, and mirror the pattern of colonial resource exploitation, where access to essential resources and services is severely restricted, while contributions to such resources are strictly imposed. However, in a system where access to NHS care is inextricably linked to both financial ability and immigration status, for some migrants, the capacity to pay does not eliminate the systemic injustice.

### Fear of surveillance and immigration enforcement

There is a profound climate of fear and mistrust among terminally ill migrants, who view healthcare engagement as a potential trigger for immigration enforcement. This fear—whether based in fact or perception—often deters individuals from seeking treatment or disclosing medical needs, worsening health outcomes and social isolation. Even in end-of-life care, the line between healthcare and border control appears blurred.

For some families stopping treatment and using healthcare was a proactive move to shield themselves from unwanted attention by the Home Office. By continuing to use the NHS, some felt other members of their family risked being identified as non-compliant with their visa requirements. For the following family, neither the terminally ill person nor their daughter felt able to access healthcare:

The family’s access to healthcare, they stopped it themselves. […] the costs were building up too much and they believed that it would draw attention to the…I don’t want to use the word illegal – unregularised status of even her daughter. […] they believed that pursuing this treatment via the NHS or via any other way will also now bring attention to the daughter’s own unregularised status. […] neither of them is accessing healthcare. (Student Social Worker – Hospice Placement)

The fear of being removed from the UK was due in part to the overlap between healthcare (including hospices) and government. With bordering activities taking place within health services, patients were unsure if seeking care would impact their immigration status:

The Home Office might push back and say, look this person is from so and so country, they can go back to their country and get help. But we all know that the standard of our healthcare here is far better […] So, it is either you break that news to these people and say, we continue to push and try, or they decide to go underground […] they stay away from the hospice […] it’s very hard for them to then believe that the hospice is a separate entity from the government. (Hospice Social Worker)

The quotation above shows a contradictory position. The speaker implied the person is staying in the UK to access high-quality care, but the converse occurred where they purposefully avoid accessing palliative care; their need for immigration security eclipses their need for healthcare access.

One frequently cited consequence of accessing NHS and hospice care was the fear of impacting immigration status. Sites of care become sites of fear and threat and, hence places to avoid or mistrust:

if there are immigration issues people are quite distrustful about any authority type of institution sort of thing because they’re worried about what might happen if people start asking questions. So, we often see people late on or not engaging as much as maybe they could. (Palliative care nurse)

The speaker draws attention to the difficult choices people may need to make about whether to seek care.

## Discussion

The data demonstrate the legal ambiguity and bureaucracy of charging regulations and practices for migrants with terminal illness. Costs are literal and figurative. They include financial payments for healthcare, emotional cost related to increased statutory surveillance and the price paid for visas and legal advice. While patients and professionals struggle to understand entitlements, uncertainty and confusion about paying for healthcare acts as a deterrent to access treatments and care. Migrants with terminal illness are therefore at risk of not having their human right met to receive the highest attainable standard of health.^27 28^ In the context of a hostile immigration environment, the opacity and complexity of rules, high charges and deeply negative impact on dying migrants could be interpreted as purposeful and punitive.

These findings resonate with international research documenting similar challenges. For instance, studies in Australia^29^ and Canada^30^ have shown that migrants and asylum seekers encounter comparable barriers to end-of-life care. Likewise, German and Nordic contexts reveal how welfare systems can either mitigate or exacerbate such inequities depending on policy design.^31^ Situating the present findings within this global evidence base highlights the universality of the issue and underscores the need for transnational policy learning.

The growth of bills and debts for terminally ill migrants reflects the cumulative financial strain described by participants. These charges may be viewed as mechanisms of control which operate not only through explicit economic exclusion, but also through governmentality^32^—the diffuse, bureaucratic strategies that produce governable subjects. In this context, uncertainty and confusion around entitlements to care function as technologies of deterrence, which render migrants internalising risk, responsibility and fear. Places of care become sites of control and migrants with terminal illness are thus exposed to biopolitics, where the right to health is undermined through a regime of opacity, delay and debt. Migrants with culturally and linguistically diverse backgrounds often rely on family caregiving and face limited access to formal palliative services. Likewise, refugees in resettlement countries experience compounded financial and emotional strain due to restricted service access and low health literacy.^33^ Globally, community-based health assets, such as peer support and social capital, can mitigate these pressures but remain underdeveloped in end-of-life contexts.^34^

The denial of palliative care is not an aberration, but a consequence of a system that governs through the withdrawal of care. Healthcare thus becomes a site where the imperative to raise revenue intersects with the logic of crimmigration^35^—the fusion of criminal and immigration control. Healthcare services are required to surveil and regulate life at the margins of legality and belonging.^36^ This calls forth theories of necropolitics, where the power to determine who dies (or is afforded a good death) becomes a mode of governance.^6 37^

The findings show that there is persistent fear of surveillance. While some healthcare professionals resist these bordering practices, often appealing to ethical norms or humanitarian principles, these acts of resistance remain fragmented. Indeed, within the context of bordering in healthcare, ethical norms and humanitarian principles *become* acts of resistance. Without dismantling the mechanisms that tie healthcare access to immigration enforcement and payments, dying migrants continue to be subject to a sovereign logic that differentiates those who can easily or problematically access palliative care.^38^ Across Europe and beyond, the integration of migrant data into health information systems has blurred the line between care and surveillance, producing new forms of ‘digital bordering’.^39 40^ These dynamics echo the experiences described by participants, where documentation checks and eligibility assessments transformed care encounters into sites of scrutiny. Such patterns are increasingly recognised as part of a global trend toward the securitisation of health governance.^41 42^

Our data reflect literature reporting healthcare staff supporting dying migrants who resist bordering practices,^43^ citing it as a contradiction to their professional ethics and moral obligations.^44^ However, the stance of individuals as isolated sites of resistance is not sufficient to systemically reframe palliative care as safe to access care, if it threatens debt or removal from the country. Palliative care, alongside other healthcare specialities, risks becoming a site of ‘crimmigration’, where bordering activities identify people accessing care and potentially criminalising them if they are in breach of visa terms.^35^

### Practice and policy implications

With increasing awareness of the financial precarity of people at end of life^11 45 46^ there is a pressing need to understand and advocate for migrants, who are at particular risk of immigration control, poverty and experiencing barriers in accessing high quality healthcare in the UK. Indeed, financial precarity should be considered part of how total pain is conceptualised, since considerable distress is generated by concerns about money.^47^

There are opportunities to address the paucity of understanding about healthcare charges. Development of a practitioner guide*,* signposting organisations, institutions and charities working in the health/palliative care and immigration sectors, would facilitate better support for terminally ill migrants including with visas and debts. This would mirror initiatives to develop ‘resource packs’ for migrants within Europe.^48^ Further, sector-specific training is needed to ensure staff understand individuals’ and families’ rights and entitlements, alongside basic immigration legislation.

### Strengths and limitations

This study foregrounds the underreported topic of bordering practices towards terminally ill people, adding to the call for greater understanding of financial precarity at end of life^9^ and on migrant health.^20^ Despite extensive efforts, no people with direct lived experience of migration and terminal illness were recruited. This reflects the marginalised and risky status of terminally ill migrants who are worried about identifying themselves to organisations. Future research should be conducted by community-based organisations that have established the trust of the migrant populations they serve, while being supported at a professional distance by academics whose direct and visible engagement could elicit responses of fear and suspicion.

## Conclusion

This paper contributes to the literature on bordering practices in healthcare,^49^ and is one of few empirical studies to report on this in palliative care,^50^ with most other work focusing on mental health, sexual health or maternity services.^51^ Our work also adds to the growing literature on financial insecurity at end of life. Although a UK-based study, the risk to people’s human rights in being unable to access appropriate high-quality healthcare is a universal concern.

Service commissioning and strategies to widen access and promote equity must be cognisant of the complex constellation of circumstances in which migrants live and die. There is a clear imperative to identify and dismantle structures which uphold and perpetuate inequality.^9^ Palliative care policies may cite marginalised groups such as minoritised ethnic communities, yet often this is not accompanied by specific recommendations or requirements to address migrants who face multiple layers of disadvantage.

## Supplementary material

10.1136/bmjopen-2025-110213 online supplemental file 1

## Data Availability

Data are available upon reasonable request.
