# Supplementary material for: How do health, social care and legal professionals support visa-dependent migrants affected by a terminal illness in the UK: a qualitative interview study
Source: BMJ Open. 2026 Sep 24;16(9):e110213. doi: 10.1136/bmjopen-2025-110213 (PMC13630090; doi:10.1136/bmjopen-2025-110213)
Supplement: Supplementary file 1 [file bmjopen-2025-110213.reviewer_comments.pdf]

PEER REVIEW HISTORY

BMJ Open publishes all reviews undertaken for accepted manuscripts. Reviewers are asked to complete a checklist review form and are provided with free text boxes to elaborate on their assessment. These free text comments are reproduced below.

ARTICLE DETAILS

Title (Provisional)

How do health, social care, and legal professionals support visa-dependent migrants affected by a terminal illness in the UK: a qualitative interview study

Authors

Sedgley, Tim; Alexander, Joanne; Macgregor, Aisha; Forbat, Liz

---

VERSION 1 - REVIEW

**Reviewer**	**1**
**Name**	**Ribi, Karin**
**Affiliation**	**Careum School of Health, part of Kalaidos University of Applied Sciences**
**Date**	**15-Feb-2026**
**COI**	**None**

This is an interesting study of an underreported topic, demonstrating the interplay between healthcare and border practices that creates unequal access to palliative and end-of-life care for migrants.

I have some comments that should be addressed by the authors.

The authors' understanding of and definition of terminal illness, and the type of migrant they are referring to, should be clarified.Type of migrant?

Inclusion criteria: How was language takein into account fpr people with lived experience? I understand that, in the end, no interviews were conducted with people who had lived experience, but were there any considerations regarding how to interview them if they did not speak English well enough?

Sampling: What was the sampling strategy for the interviewees?

Participant characteristics: What were the characteristics of the interview participants expect their professional background (e.g. Age, gender, years of professional experiences?)

Interview guide: The authors mention "….how people navigate treatment, work, and visa requirements, how patients/families manage financially…." Here it is not clear who is meant by people: those with lived experience/patients or professionals.

Minor:

I suggest moving the paragraph on the research team's reflexivity after the paragraph on the analysis. This would allow the reader to better understand the reflexive analytical process by having the team description directly afterwards.

The note that the interviews were semi-structured should appear in the methods not the findings.

COREQ:

1. The initials of the interviewers are not included on page 4.

10. Sampling strategy is not described on page 4 as indicated.

16. Description of the sample incomplete on page 5.

**Reviewer**	**2**
**Name**	**Giannitrapani, Karleen**
**Affiliation**	**VA Center for Innovation to Implementation**
**Date**	**19-Jun-2026**
**COI**	**none**

Dear Authors,

Title: How do health, social care, and legal professionals support visa-dependent migrants affected by a terminal illness in the UK: a qualitative interview study

Thank you for the opportunity to review this manuscript regarding the barriers to care and support faced by terminally ill migrants in the UK health system. This is an important and relevant topic as healthcare disparities are rampant internationally with migrants often experiencing the most harm. Please see the comments below for an effort to improve the manuscript.

Major overall comment:

The focus on the end of life care for migrants is an important contribution to existing literature because of the unique stresses that it poses. I have no major concerns with the methods.

Comments by section:

Background: The background provided appropriate context for the difficulties faced by terminally ill migrants and addressed relevant changes in the National Health Service that underlie these difficulties. Since the study did not consult any terminally ill migrants, the background should include context for the professionals who are interviewed. For example: What are the existing protocols for treating terminally ill migrants for health/social care professionals? What does access to lawyers look like for these patients?

Methodology: The guiding questions at the beginning are helpful, but given the extensive responses in the Findings, it would be helpful to know what kinds of follow up questions were asked during the interviews. Follow up interviews were also conducted, and I am curious about why they were necessary/ what they were asked/ what insights were added? Were the same questions asked by role? Did any differences emerge by role?

Findings: The specific quotations outlining situations these professionals had encountered were very powerful. Citing the professions of the individuals who responded helps explain the extent to which the experts are struggling to support this community. It would be helpful to have the context by role of who is making the quote.

Discussion: Ultimately, the discussion points to the fact that health care professionals' individual acts of resistance in providing care is not enough and that a systematic shift has to occur to properly support terminally ill migrant communities. In order to support this claim more ensure that all of the themes are addressed and enhanced through the literature. This section could benefit from more explicit continuity with the Findings section. This also could benefit from literature addressing this issue in other international contexts.

Strengths and Limitations: It is a notable limitation that no individuals with lived experience participated in the study, but it was explicitly and transparently explained.

It was great to see that along with the gift card provided to the participants, a list of support organizations was given, it shows dedication to the impacted community.

VERSION 1 - AUTHOR RESPONSE

REVIEWER ONE: Prof. Karin Ribi,

Comment: This is an interesting study of an underreported topic, demonstrating the interplay between healthcare and border practices that creates unequal access to palliative and end-of-life care for migrants. I have some comments that should be addressed by the authors.

Response: thank you for your positive comments about the paper

Comment: The authors' understanding of and definition of terminal illness, and the type of migrant they are referring to, should be clarified. Type of migrant?

Response: We have now included these details in the methods.

Comment: Inclusion criteria: How was language take in into account for people with lived experience? I understand that, in the end, no interviews were conducted with people who had lived experience, but were there any considerations regarding how to interview them if they did not speak English well enough?

Response: we have added a brief note about this into the paper's inclusion criteria section to explain that we had information leaflets about the study ready to be sent in a range of languages. We also had a budget for interpreters should people prefer/need to conduct the interview in a language that the team did not speak. We also had PPIE members input into the

participant materials to ensure participant and recruitment materials were accessible and used lay language.

Comment: Sampling: What was the sampling strategy for the interviewees? Participant characteristics: What were the characteristics of the interview participants expect their professional background (e.g. Age, gender, years of professional experiences?)

Response: We did not collect details of participants age, gender or years of professional experience. Concerns about compromising anonymity meant that collecting and then reporting such detail risked identifying participants. However while still guarding participant anonymity we have added some more details of their professional background in Table 1 (note: the intext reference to Table 1 is track-changed, but not the addition of the table to aid readability for the reviewer). The sampling strategy has been expanded in the methods section to indicate the methods used to identify participants.

Comment: Interview guide: The authors mention "....how people navigate treatment, work, and visa requirements, how patients/families manage financially...." Here it is not clear who is meant by people: those with lived experience/patients or professionals.

Response: we have clarified this in text earlier in the sentence to clarify these were questions asked to the range of participants

Comment: Minor: I suggest moving the paragraph on the research team's reflexivity after the paragraph on the analysis. This would allow the reader to better understand the reflexive analytical process by having the team description directly afterwards.

Response: thanks for this suggestion. We have moved the paragraph as suggested.

Comment: The note that the interviews were semi-structured should appear in the methods not the findings.

Response: we have removed the phrase 'semi-structured' from the findings and ensured it is in the methods.

Comment: COREQ: 1. The initials of the interviewers are not included on page 4.

Response: these have now been added

Comment: COREQ10. Sampling strategy is not described on page 4 as indicated.

Response: this has now been added

Comment: COREQ16. Description of the sample incomplete on page 5.

Response: this has now been addressed by Table 1 on p7.

REVIEWER TWO: Dr. Karleen Giannitrapani,
Comment: Major overall comment: The focus on the end of life care for migrants is an important contribution to existing literature because of the unique stresses that it poses. I have no major concerns with the methods.

Response: Thank you for your positive response to the paper

Comment Background: The background provided appropriate context for the difficulties faced by terminally ill migrants and addressed relevant changes in the National Health Service that underlie these difficulties. Since the study did not consult any terminally ill migrants, the background should include context for the professionals who are interviewed. For example: What are the existing protocols for treating terminally ill migrants for health/social care professionals? What does access to lawyers look like for these patients?

Response: We have inserted a new sentence into the background section attending to the question of what palliative care services are available to terminally ill migrants. However, this is also one of the findings – indicated in the opening sentence of the section '*Legal ambiguity and bureaucracy'*. *With regard to legal access, we have inserted a new sentence into the findings under the same subsection indicating* what our interviewees told us (p8).

Comment Methodology: The guiding questions at the beginning are helpful, but given the extensive responses in the Findings, it would be helpful to know what kinds of follow up questions were asked during the interviews. Follow up interviews were also conducted, and I am curious about why they were necessary/ what they were asked/ what insights were added? Were the same questions asked by role? Did any differences emerge by role?

Response: follow-up questions were typical of the kind in semi-structured interviews, where the prompts seek further depth, clarification or curiosities based on what the interviewee says. These were therefore quite varied.

Only one follow-up interview was conducted as the interviewee requested more time to reflect on and unpack a dynamic changing situation (they were speaking about a patient who was currently using the service, rather than someone who had already died.) We have added a sentence about this into the paper (under 'data collection') to clarify this for the reader.

We have provided the interview topic guide so readers can see the kind of initial prompt questions used. Since this paper reports data pertaining to professionals, we have uploaded only those questions (not the unused ones for patients).

Comment Findings: The specific quotations outlining situations these professionals had encountered were very powerful. Citing the professions of the individuals who responded helps explain the extent to which the experts are struggling to support this community. It would be helpful to have the context by role of who is making the quote.

Response: Each quotation in the paper was accompanied by a note about the professional's identity. Due to the small numbers working in this specialist field together with the seemingly contentious nature of the topics we are covering, we have been conscious not to give too much information in publications related to roles, job titles, locations and context. This is in an effort to protect the identities of participants as far as possible. We hope this provides Reviewer Two with some clarity on our reporting decisions, especially around role context.

Comment Discussion: Ultimately, the discussion points to the fact that health care professionals' individual acts of resistance in providing care is not enough and that a

systematic shift has to occur to properly support terminally ill migrant communities. In order to support this claim more ensure that all of the themes are addressed and enhanced through the literature. This section could benefit from more explicit continuity with the Findings section. This also could benefit from literature addressing this issue in other international contexts.

Response: The discussion has been more clearly linked with each of the findings subsections, and international literature/links added.

Comment Strengths and Limitations: It is a notable limitation that no individuals with lived experience participated in the study, but it was explicitly and transparently explained.

Response: thank you.

Comment It was great to see that along with the gift card provided to the participants, a list of support organizations was given, it shows dedication to the impacted community.

Response: thank you.
