# Supplementary material for: How do health, social care and legal professionals support visa-dependent migrants affected by a terminal illness in the UK: a qualitative interview study
Source: BMJ Open. 2026 Sep 24;16(9):e110213. doi: 10.1136/bmjopen-2025-110213 (PMC13630090; doi:10.1136/bmjopen-2025-110213)
Supplement: Supplementary file 2 [file bmjopen-16-9-s001.docx]

Interview topic guide

*Prompts adjusted depending on interviewee’s field of work.*

**Introduction**

- Could you start by introducing yourself (e.g., What sector you’re in, what your role is, who your client group is etc)?

**Your experiences of supporting families on UK visas**

- Can you describe how your role and the work you do relates to supporting families on UK visas?
- Do you support individuals/families on particular visa types or with particular immigration status?
- Can you talk through your experiences of supporting people dependent upon visas for their residency in the UK who are facing terminal illness?
- What are some of the common issues you support individuals and families with?
- What are the challenges experienced by people on UK visas when they or their dependent family members face terminal illness?
- What are the key concerns you have for service users in these circumstances?
- How does your service and others respond to families and intervene in issues related to terminal illness and UK visas?
- Can you give example of effective service responses?
- Can you give examples ineffective or unhelpful responses/interventions?

**Balancing health, work, & visa requirements**

- In your experience, how might people manage residency in the UK when affected by terminal illness?
- Does immigration status have to be re-considered? (e.g., to stay on a visa, to apply for an alternative type of visa, or to leave the country etc?)
- In your experience, what  are some of the strategies people develop to manage health, meet financial needs/make an income, and comply with visa requirements?
- How are people supported to maintain residency in the UK when they or a dependent family member are ill?
- Are your clients entitled to work? Are there restrictions on working due to visa type?
- If relevant, do the clients you work with feel obliged to continue working while ill ? If so, what is the impact of this for families?
- Are your clients entitled to access benefits?

**Managing finances**

One of the main aims of our study is to understand how people on UK visas cope financially in the face of a terminal illness.

- How do your clients manage financially?
- If relevant, do your clients change their working practices due to illness (e.g., change and/or reduce working hours, or terminate work?)? If so, what are the impacts of this?
- Are you aware of your clients experiencing financial insecurity or reduced household income due to illness?
- How are service users managing on reduced budgets? (e.g., have other family members taken on additional work etc?)
- What financial support is available for people affected by illness on UK visas? Who can/do you signpost/refer your clients to?

**Accessing health care & legal support**

- What are the needs families have in accessing healthcare?
- Are there obstacles for the families you work with in accessing health/social care or any other services?
- Do your clients ever experience restrictions to healthcare due to their immigration/visa status?
- Do they ever have to pay for healthcare? If so, do you have a sense of the impacts of this on your clients?
- Do you know how your clients are navigating immigration law?
- What are the central needs families have in navigating immigration law and accessing legal support?
- Other than your service, are there any other organisations/professionals your clients access support from?  If so, who are they? / What do they support with? / How frequently?

**Improving policy & practice**

- What policy and/or practice could be improved to better help people in such circumstances in the future?
- What do you think are some of the helpful and unhelpful narratives/discourses relating to migrants and UK visas operating in public and professional spheres?
